# Effects of altitude on chronotype orientations in relation to cardiorespiratory and hematological quantities of college students in Ethiopia

**DOI:** 10.1371/journal.pone.0219836

**Published:** 2019-07-17

**Authors:** Efrem Kentiba, Mala George, Soumitra Mondal, D. Mathi Vanan

**Affiliations:** 1 Department of Sports Science, Arba Minch College of Teachers’ Education, Arba Minch, Ethiopia; 2 Department of Sports Science, Mekelle University College of Natural and Computational Sciences, Mekelle, Ethiopia; 3 Department of Biochemistry, School of Medicine, Division of Biomedical Sciences, Mekelle University, Mekelle, Ethiopia; University of Brasilia, BRAZIL

## Abstract

**Background:**

The mechanism by which Ethiopians adapt to altitude is quite unique compared to other Highlanders with respect to increased oxygen saturation of hemoglobin. Although the effects of altitude on cardiorespiratory and hematological quantities on athletics performances are well known, but there is little information about its underlying effect on chronotype orientations.

**Methods:**

In this cross-sectional study 60 male college students with mean age 20±1.3 years from high and low altitude regions living in a tropical setting in Ethiopia were included. The participants’ chronotype was determined using the self-administered Horne and Ostberg Morningness-Eveningness Questionnaires (MEQ). Measurements and estimations of hematological and cardiorespiratory parameters were performed from 7:00–9:00 AM, East African time zone, in order to minimize any variations that might occur in the course of the day. A multivariate binary logistic regression model was fitted to analyze the underlying chronotype predictors.

**Results:**

28 (93.9%) of participants from high altitude were mainly intermediate type (I-type) dominant with (MEQ = 42–58). While, 16 (55.2%) of participants from low altitudes were morning type (M-type) dominant chronotype with (MEQ = 59–69). Our main finding confirmed that altitude is an independent predictor of chronotype orientations of the participants (p<0.015). Thus, the results of the multivariate analysis seem to indicate that, participants from low and high altitudes may be uniquely oriented towards either M-type or I-type chronotype respectively (adjusted odds ratio [AOR] 4.772, 95% CI = 3.748–4618458). However, no significant difference on cardiorespiratory and hematological quantities between I-type and M-type chronotype of students from low altitude living in the same setting was reported (p > 0.05).

**Conclusion:**

Our finding, reported for the first time that, the human chronotype varies according to the altitude, with no underlying effect of cardiorespiratory and hematological quantities.

## Introduction

Altitude adaptation in humans is an instance of evolutionary modification in certain populations, like the Tibetans, Andean inhabitants and Ethiopians [1]. This is perhaps due to their migration to the highland was relatively early [2]. The adaptation to high altitude among different Highlanders also arose independently of convergent evolution [3]. The analysis of specific genes that influence adaptation to high altitude seems to be expressed highly among Ethiopians compared to other Highlanders [4]. These genes are known to produce hypoxia induced factors known to regulate production of red blood cells and oxygen saturation of hemoglobin [5]. However, a sustained exposure to hypoxia may alter body composition [6], raise stress hormones [7], affects the volume of maximum oxygen consumption (Vo_2_Max) and sleep patterns [8–10].

Further studies have highlighted differences between populations living in high and low altitudes based on morphophysiological characteristics [11,12]. Thus, people living at high altitudes have been reported to be thinner and shorter than those from the sea level. This is due to the effects of altitudes on oxygen saturation of hemoglobin [5], Vo_2_Max, stress hormones [7] and blood compositions and circulation [13]. Hence, the irreversible, long-term physiological responses are associated with heritable behavioral changes in chronotype orientations [3,14,15]. Although the effects of altitude on cardiorespiratory and hematological quantities on athletics performances are well known [11–15], but there is little information about its underlying effect on chronotype orientations.

Chronotype refers to individual’s time-of-day preferences of activities that can be classified as ‘‘M = morning type”, ‘‘E = evening type “and “I = intermediate types” [16–18]. However, peoples' preference of activity schedules may not be possible to match with their chronotype orientations [16,19,20]. This is in spite of the reported impact of a person’s chronotype orientation on their response to exercise [21,22], academic achievement and other activities [23–25]. Furthermore, recent studies have reported that the observed chronotype in athletes may be different to that of non-athletes [26–30] and varied either by longitudes or latitudes [31]. The underlying influence of longitudes and latitudes on chronotype orientations has been related to scotopic periods (i.e., the time interval between sunset and sunrise) and photopic periods (i.e., the time interval between sunrise and sunset) [32–34]. Therefore, the exposure to bright sunlight may affect the phase position of the main sleep episode, leading to different sleep-wake patterns [35].

In our previous study, we reported that the chronotype preferences of college students may vary according to their altitude backgrounds [36]. However, the underlying parameter by which altitudes may influence chronotype orientations is still unknown. Hence, cross-sectional study design was used to assess cardiorespiratory and hematological quantities of 60 untrained male college students from high and low altitude living in tropical settings of Ethiopia. To associate the influence of altitude on chronotype orientations in relation to cardiorespiratory and hematological quantities, a multivariate binary logistic regression analysis was used. Thus, the outcome of this study may be important to exercise physiologists, coaches and athletes while scheduling training and competition for participants from either high or low altitude backgrounds living in tropical settings.

## Materials and methods

### Study setting and ethical approval

Ethical approval was obtained from Mekelle University College of Health Sciences; Health Research Ethics Review Committee (HRERC) with Ref. ERC 1078/2017 dated 26/06/2017 and conducted in accordance with the declaration of Helsinki. In addition a written consent to participate in the study was obtained from participants. The study was conducted in Arba Minch town South-West Ethiopia, with an elevation of 1286 meters above sea level [37,38], which is categorized as low altitude tropical setting [39]. It is located at 6.0206°N and 37.5641°E, latitude and longitude coordinates with an average annual temperature of 25.2°C, (average high and low temperature of 28.7°C and 21.8°C respectively) [36].

### Study design and participants

**Fig 1:**
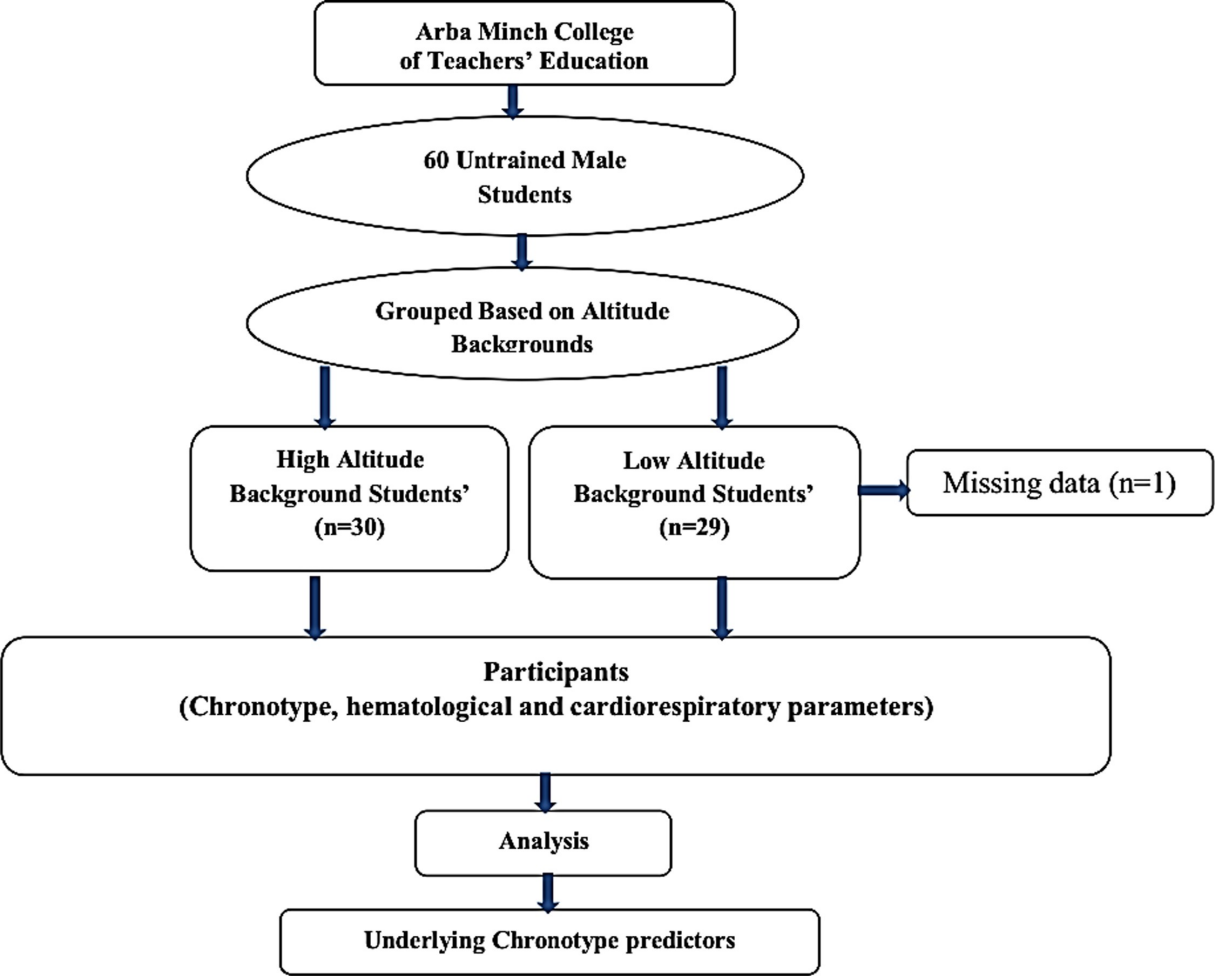
Study design and data collection procedures.

A cross-sectional study design was used, to assess the chronotype, cardiorespiratory and hematological quantities of 60 male college students with mean age 20±1.3 years from high and low altitude regions living in tropical settings of Ethiopia (Fig 1). The number of participants was based on a formula for sample size of the mean [40]; assuming 95% confidence interval, 5% margin of error and 0.1 standard deviation obtained from previous studies [41,42]. A self-reported demographic questionnaire was used to determine, among other things the places of their origin and growth before joining the college within the study setting. Therefore, based on the altitude classification by the Canadian Academy of Sport and Exercise Medicine (CASEM) [39], participants who originated and grew up from areas between 500–2000 meters above sea level were grouped under low altitude backgrounds. While those from areas above 3000 meters above sea level were grouped under high altitude backgrounds [36,39]. However, participants who reported to be from neither high nor low altitude backgrounds were excluded from the study. Based on the information obtained, participants were grouped either to high (n = 30) or low altitude backgrounds (n = 29) with (n = 1) missing. Then after, data about chronotype orientations, hematological and cardiorespiratory parameters were obtained. The participants’ chronotype was determined using the self-administered Horne and Ostberg Morningness-Eveningness Questionnaires (MEQ) [43].

### Procedures

All students participating in the study had no any preceding intense exercise, declared as healthy and in good physical condition. Measurements and estimations of hematological and cardiorespiratory parameters were performed from 7:00–9:00 AM, East African time zone, in order to minimize any variations that might occur in the course of the day [41,44–47]. Vo_2_max as an indicator of cardiorespiratory endurance (CRE) [48], was estimated using maximal exercise test (20 meter multistage shuttle run test) or beep test [49–50]. The test has been shown to be an accurate method to estimate Vo_2_Max in young adults with (r = 0.9) [51]. The test was employed as outlined by the American College of Sports Medicine [52]. Generally, it was taken at 8.5 km/hr (level^-1^) and increased by 0.5km/hr at each level. The result was obtained by using online beep test calculator (BTC) based on the number of shuttles attained at each level. The calculator appears to be accurate within 0.1 ml/kg/min of the published values [53].

Blood sampling was done according to the procedures explained by Simundic et al. 2017. Participants were left to sit for 15 minutes prior to sampling. 5 ml venous blood was drawn from the ulnar vein of the non-dominant hand using a 20 G x 1½″–0.9 x 40 mm syringe after application of a tourniquet and cleansing the site. The blood was introduced into a tube with Ethylene Di-amine Tetra Acetate (EDTA) to determine the concentration of erythrocytes, leukocytes and thrombocytes using a hematology analyzer (BC-3000Plus Mindray Medical, Andheri East, Mumbai, Maharashtra India) [54].

### Data analysis

All data were tested for normality using the Pearson normality test. Descriptive statistics were expressed either as mean ± standard deviation or frequency (proportion) for continuous and categorical variables respectively. To associate the influence of altitude on chronotype orientations in relations to cardiorespiratory and hematological parameters, a multivariate binary logistic regression analysis was conducted. Independent sample t-test was used to compare the mean morningness-eveningness questionnaire results (MEQR), V^.^o_2_Max, body mass index (BMI) and hematological parameters between students from high and low altitude backgrounds. Similarly, we used the above test to compare MEQR of V^.^o_2_Max and hematological quantities between intermediate type (I-type) and morning type (M-type) chronotype orientations of students from low altitude backgrounds. All statistical analyses were performed using IBM-SPSS version 20 (IBM, Armonk, NY, United States of America). All reported p-values are two tailed and confidence intervals are calculated at 5% alpha value.

## Results

**Fig 2:**
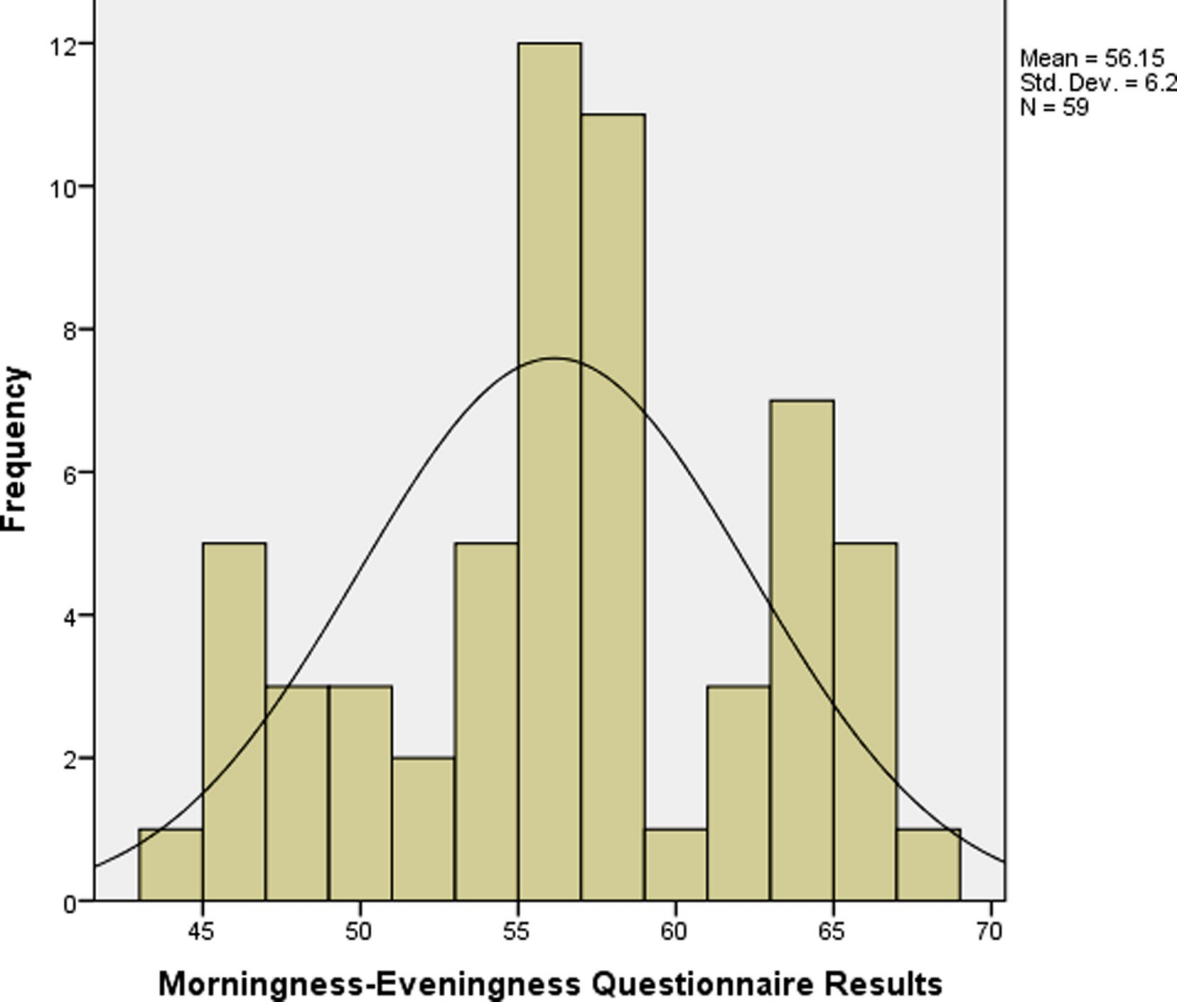
MEQ scores of students from high and low altitudes living in tropical settings.

**Fig 3:**
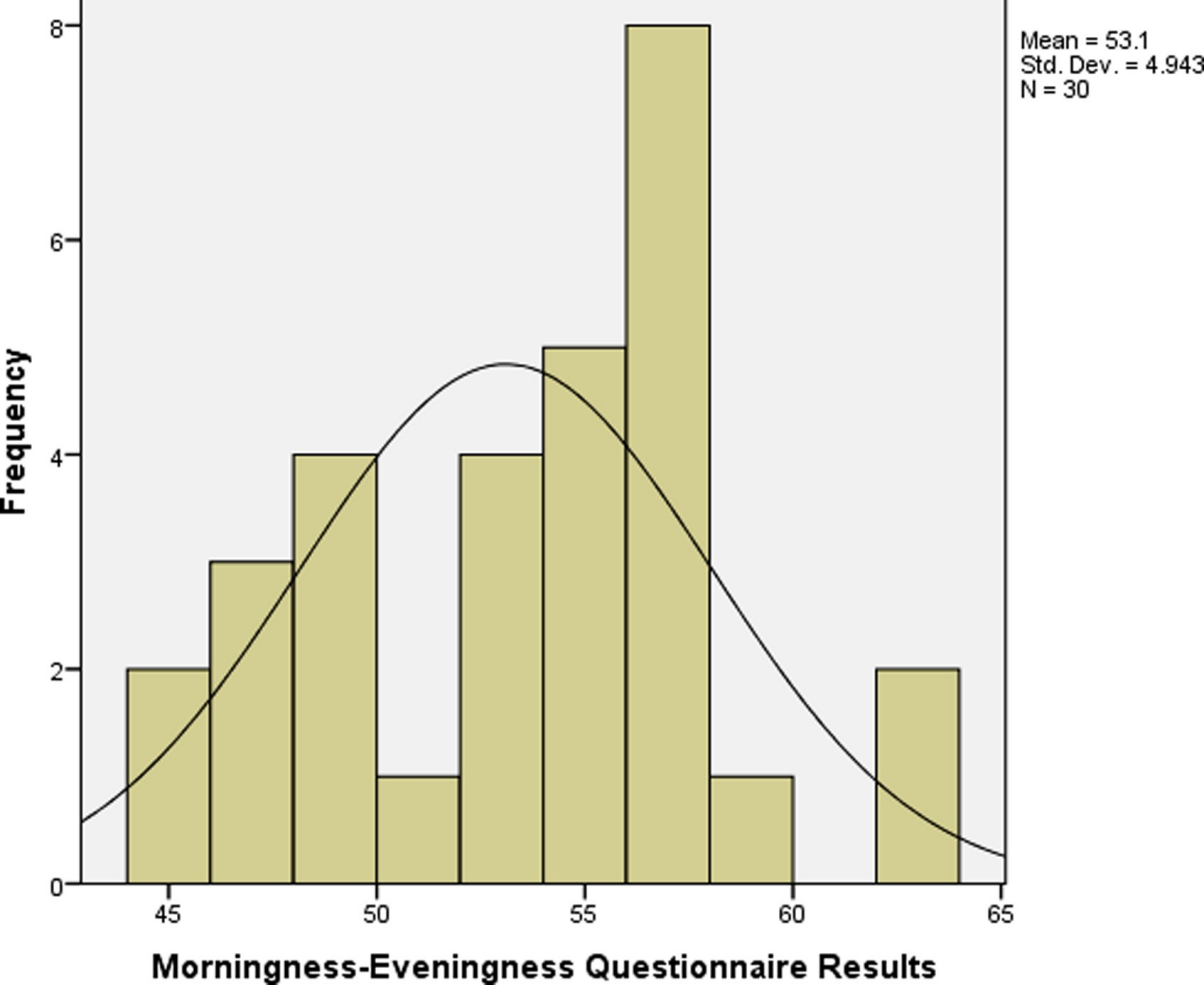
MEQ scores of studens from high altitude backgrounds.

**Fig 4:**
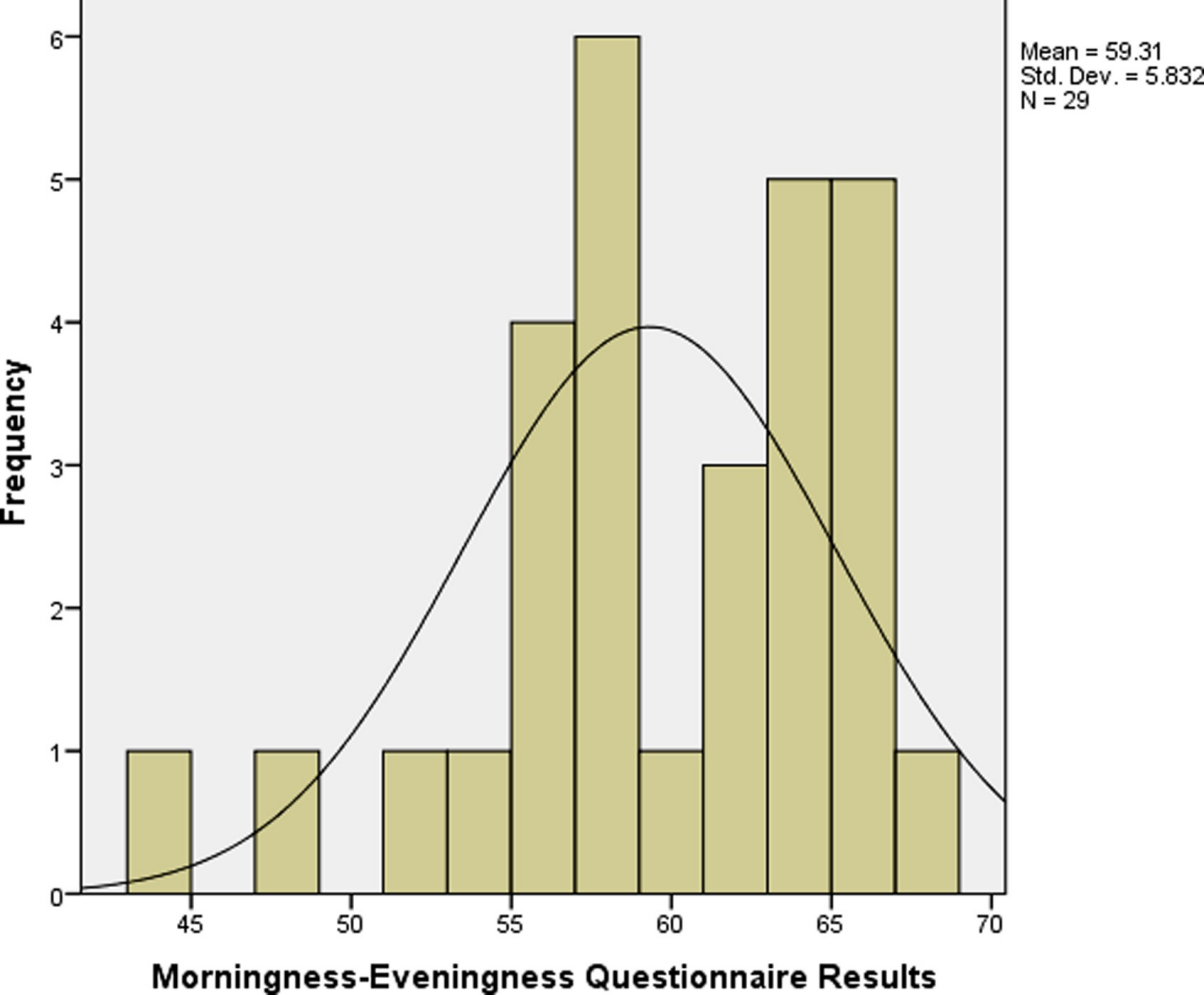
MEQ scores of students from low altitude backgrounds.

No between-group differences (Age, height, body mass and BMI) existed at baseline, so the groups were well matched at entry level (Table 1). The chronotype preferences of participants from high compared to low altitudes indicated significant differences (P<0.001). Thus, 28 (93.9%) of participants from high altitude were mainly (I-type) dominant chronotype (MEQ = 42–58). While 16 (55.2%) of participants from low altitudes were (M-type) dominant chronotype (MEQ = 59–69) (Figs 2–4).

**Table 1:** Comparison of demographic, hematological, cardiorespiratory and chronotype orientations between participants from high and low altitudes (n = 59).

Variables		High Altitude Backgrounds (n = 30) (Mean ± SD)	Low Altitude Backgrounds (n = 29) (Mean ± SD)	*p-value*
Age		19.93±1.45	20.28±1.16	0.319
Height		1.68±0.05	1.70±0.08	0.285
Body mass		61.70±4.70	61.91±4.82	0.864
BMI		21.49±1.95	22.05 ± 1.57	0.223
Chronotype preferences		I-type = 28 (93.9%)	M-type = 16 (55.18%)	
		MEQR = 53.10±4.94	MEQR = 59.31±5.83	0.001
Cardiorespiratory Parameters (Vo_2_Max)		51.84 ± 7.64	47.25 ± 9.41	0.044
Hematological quantities				
	HGB in g/dL	16.43 ± 1.19	15.69 ± 0.91	0.010
	RBC in (x10^6^ / μL)	5.33 ± 0.31	5.15 ± 0.32	0.034
	HCT in %	49.16 ± 2.96	47.20 ± 2.49	0.008
	MCV in fL	92.45 ± 3.69	91.8 ± 3.17	0.501
	PLT in (x10^3^ / μL)	234.37 ± 39.58	254.06 ± 57.96	0.132
	WBC in (x10^3^ / μL)	7.01±2.00	5.74 ±0.70	0.015

Vo_2_Max = volume of maximum O_2_ consumption in ml/(kg*min), MEQR = MEQ results, BMI = body mass index, HGB: hemoglobin count, RBC: red blood cell count, HCT: hematocrit, MCV: mean corpuscular volume, PLT: platelet, WBC: white blood cell count

The mean cardiorespiratory and hematological quantities of participants from high compared to low altitudes also indicated significant differences except for MCV and PLT (p< 0.05). However, there was no significant differences in the mean hematological and cardiorespiratory quantities between I-type and M-type chronotype orientation of participants from low altitude backgrounds (p > 0.05) Tables 1 and 2.

**Table 2:** Mean hematological and cardiorespiratory parameters between I-type and M-type chronotype orientations of participants from low altitudes backgrounds (n = 29).

Variables		Low Altitude Backgrounds		
		I-type (n = 13) (Mean ± SD)	M-type (n = 16) (Mean ± SD)	*p-value*
Cardiorespiratory parameters (Vo_2_Max)		49.09**±**10.09	45.75**±**8.86	0.352
Hematological quantities				
	HGB in g/dL	15.85±0.62	15.57±1.10	0.413
	RBC in (x10^6^ / μL)	5.17±0.30	5.13±0.34	0.757
	HCT in %	47.47±1.75	46.97±3.01	0.600
	MCV in fL	92.06±3.43	91.66±3.04	0.738
	PLT in (x10^3^ / μL)	246.53±46.23	260.18±66.87	0.538
	WBC in (x10^3^ / μL)	5.70±0.80	5.78±0.64	0.765

Vo_2_Max = volume of maximum O_2_ consumption in ml/(kg*min), HGB: hemoglobin count, RBC: red blood cell count, HCT: hematocrit, MCV: mean corpuscular volume, PLT: platelet, WBC: white blood cell count

The predictor variables of chronotype orientation of students from varied altitude backgrounds are given in Table 3. The only significant predictor of the chronotype orientations was the altitude background of the participants (p<0.015). Thus the results of the multivariate analysis seem to indicate that, participants from low and high altitudes may be uniquely oriented towards either be M-type or I-type chronotype respectively (AOR 4.772: 95% CI = 3.748–4618458).

**Table 3:** Predictive parameters of chronotype orientations among students from high and low altitude backgrounds (n = 59).

Predictive variables		B	β(SE)	Exp (B) AOR (95% C. I)	*p-value*
Age		-0.172	0.425	.842(.366–1.935)	0.686
BMI		-0.147	0.151	0.863 (0.642–1.161)	0.330
Altitude Backgrounds		8.471	3.491	4.772 (3.748–4618458)	0.015
Cardiorespiratory parameters (Vo_2_Max)		.-0.048	0.054	.953 (.857–1.060)	0.375
Hematological quantities					
	HGB in g/dL	-7.025	3.745	.001 (.000–1.370)	0.061
	RBC in (x10^6^ / μL)	-102.843	72.972	.000 (.000–281826955801916544)	0.159
	HCT in %	14.232	8.722	1517237.565 (.057–40276307899954)	0.103
	MCV in fL	-6.035	4.300	.002 (.000–10.950)	0.160
	PLT in (x10^3^ / μL)	0.017	0.013	1.017(.992–1.042)	0.193
	WBC in (x10^3^ / μL)	0.176	0.408	1.192(.535–2.654)	0.667
Constant		503.004	40194.853	2.831E+218	0.990

Variable(s) entered: Age, BMI, Blood Group, Altitude backgrounds, Vo2Max, HGB, RBC, HCT, MCV, PLT and WBC (HGB: hemoglobin count, RBC: red blood cell count, HCT: hematocrit, MCV: mean corpuscular volume, PLT: platelet, WBC: white blood cell count, Vo_2_Max: volume of maximum oxygen consumption, B: unstandardized regression coefficient, β(SE): standardized regression coefficient, AOR: adjusted odds ratio

## Discussion

This is the first study to our knowledge that investigated the effects of altitude on chronotype orientations in relation to cardiorespiratory and hematological quantities of students from high and low altitude backgrounds living in a tropical setting. Our main finding confirmed that altitude is an independent predictor of chronotype orientations of the participants [36].

Based on the cited studies [55–62] one might hypothesize that the effects of acclimatization on sleep are altitude dependent. Early, studies have suggested that a pronounced sleep fragmentation was a characteristic change occurring with exposure to hypoxia [63]. As suggested previously by Ashkenazi et al 1982, hypoxia may act as real phase-shift inducer of the circadian system [64]. This effect may be related to a delayed phase of sleep-wake cycle. Consequently, we found that participants from low and high altitudes living in a tropical setting may be uniquely oriented towards either be M-type or I-type chronotype respectively. Since, increase in sleep onset latency (SOL) after hypoxic exposure at high altitude seems to depend on the evening decline of core body temperature and plasma melatonin [65–67]. Therefore, our finding is consistent with the study by, Coste et al., 2004a that significant negative association between the SOL and the age-correlated Horne & Ostberg score reflects a close relation between an elevated morningness preference and short SOL. However, further studies are required to better quantify the effects of different levels of altitude on sleep in persons of both sexes and of various ages and to elucidate the underlying physiological mechanisms.

Most surprisingly, we did not report E-type dominant chronotype orientations, despite the young adults participating in the study. Considering that changes from M-type to E-type take place according to age, with higher prevalence of M-type occurring during childhood and higher prevalence of E-type during adolescence [23,68,69]. The adolescence age group is known to poorly tolerate altitude related stresses [8–10], which may affect their sleep patterns. Thus, in our study, the absence of E-type chronotype might be obscured by the unique evolutionary adaptation to altitude by the participants. Our finding further reported no significant difference on Vo_2_Max and hematological quantities between I-type and M-type chronotype among students from low altitude living in a tropical setting. However, previous studies reported a better VO_2_max in E-types than M-types [68,70–72]. This might be reflective of better aerobic participation in energy metabolism during the evening time, leading to both mental and physical activeness among E-type than other chronotype [16,36,73]. Although our finding is not consistent with previous studies, but this might be due to absence of E-type among compared chronotype orientations. Furthermore, participants in our study are homogenous in their altitude backgrounds (low only) and may similarly adapt to increased blood plasma and oxygen volume.

Evidence supports the notion that performance can be improved if individuals are matched with their preferred chronotype [17,36]. Our finding that human chronotype varies according to altitude may be useful for exercise physiologists, coaches and athletes while either scheduling training or competition for participants. Since, altitude effects on blood compositions and circulation may lead to an altered cardiorespiratory and hematological quantities [13]. Thus, the strength of this study is that we evaluated if the above underlying parameters of students from varied altitude background that might influence their chronotype orientations. However, students from high altitude background were majorly I-type dominant and we could not compare their chronotype orientation in relation to cardiorespiratory and hematological quantities. Furthermore, it would be interesting to assess the underlying effect of altitude on chronotype of participants from low living in high altitude in order to give bidirectional relationship. Whether our findings indicate the uniqueness of Ethiopians to altitude adaptation in tropical setting in relation to chronotype orientation needs further investigation.

### Conclusion

Our finding, reported for the first time that, the human chronotype varies according to the altitude, with no underlying effects of cardiorespiratory and hematological quantities.

## Supporting information

S1 File Raw data (Demographic and cardiorepiratory quantities). (XLSX)

S2 File Raw data (Hematological quantities). (XLSX)
